# Long-Lived Bright Red Emitting Azaoxa-Triangulenium Fluorophores

**DOI:** 10.1371/journal.pone.0063043

**Published:** 2013-05-07

**Authors:** Badri P. Maliwal, Rafal Fudala, Sangram Raut, Rutika Kokate, Thomas J. Sørensen, Bo W. Laursen, Zygmunt Gryczynski, Ignacy Gryczynski

**Affiliations:** 1 Center for Commercialization of Fluorescence Technologies, Department of Molecular Biology and Immunology, University of North Texas Health Science Center, Fort Worth, Texas, United States of America; 2 Department of Molecular Biology and Immunology, University of North Texas Health Science Center, Fort Worth, Texas, United States of America; 3 Nano-Science Center and Department of Chemistry, University of Copenhagen, København, Denmark; 4 Departments of Physics and Astronomy, Texas Christian University, Fort Worth, Texas, United States of America; 5 Department of Cell Biology and Anatomy, University of North Texas Health Science Center, Fort Worth, Texas, United States of America; CNR, Italy

## Abstract

The fluorescence lifetimes of most red emitting organic probes are under 4 nanoseconds, which is a limiting factor in studying interactions and conformational dynamics of macromolecules. In addition, the nanosecond background autofluorescence is a significant interference during fluorescence measurements in cellular environment. Therefore, red fluorophores with longer lifetimes will be immensely helpful.

Azaoxa-triangulenium fluorophores ADOTA and DAOTA are red emitting small organic molecules with high quantum yield, long fluorescence lifetime and high limiting anisotropy. In aqueous environment, ADOTA and DAOTA absorption and emission maxima are respectively 540 nm and 556 nm, and 556 nm and 589 nm. Their emission extends beyond 700 nm. Both probes have the limiting anisotropy between 0.36–0.38 at their absorption peak. In both protic and aprotic solvents, their lifetimes are around 20 ns, making them among the longest-lived red emitting organic fluorophores. Upon labeling of avidin, streptavidin and immunoglobulin their absorption and fluorescence are red-shifted. Unlike in free form, the protein-conjugated probes have heterogeneous fluorescence decays, with the presence of both significantly quenched and unquenched populations. Despite the presence of significant local motions due to a flexible trimethylene linker, we successfully measured both intermediate nanosecond intra-protein motions and slower rotational correlation times approaching 100 ns. Their long lifetimes are unaffected by the cell membrane (hexadecyl-ADOTA) and the intra-cellular (DAOTA-Arginine) localization. Their long lifetimes also enabled successful time-gating of the cellular autofluorescence resulting in background-free fluorescence lifetime based images.

ADOTA and DAOTA retain a long fluorescence lifetime when free, as protein conjugate, in membranes and inside the cell. Our successful measurements of intermediate nanosecond internal motions and long correlations times of large proteins suggest that these probes will be highly useful to study slower intra-molecular motions and interactions among macromolecules. The fluorescence lifetime facilitated gating of cellular nanosecond autofluorescence should be of considerable help in *in vitro* and *in vivo* applications.

## Introduction

Use of fluorescence in biomedical and biological sciences is ubiquitous due to its simplicity, high sensitivity, versatility, fast response time and a continuously expanding probes base. For example in diagnostics, fluorescence based assays have largely replaced use of radioactivity. Similarly, fluorescence has played a large role in our understanding of whole range of biochemical and biological processes at molecular and cellular level. Study of gene expressions and sequencing, cellular imaging, localization and transport to tissues and organs in whole body are few examples [1]–[5]. One of the problems when working with cells, tissues and other biological material is the presence of intrinsic autofluorescence that decreases towards near infrared (NIR) region of the spectrum. Equally important is tissue transparency, which is very low in ultraviolet (UV) and near-UV part of the spectrum and increases towards red and NIR. This need to minimize autofluorescence and use the red and NIR “open window“ has driven the fluorescent probe development. The basic criteria have always been that probes should be small when compared to macromolecules, photostable, of high quantum yield and large molar extinction coefficient leading to high photon flux. However, as per Strickler-Berg law [6] in case of organic fluorescent molecules the large molar extinctions also result in short fluorescence lifetimes. Almost all of newer bright and large molar extinction coefficient red and NIR organic fluorescent probes have lifetimes in the few ns range [7]. Since background cellular autofluorescence has similar decay profile as most of the available red and NIR probes, it is a source of serious interference for measurements in such environment.

Similarly, the nanosecond fluorescence lifetime limits the window available to monitor very important macromolecular conformational motions, which take place over a much larger time scale [8]–[10]. Also important are Interactions among proteins and their interactions with other macromolecules such as DNA, RNA and membranes that play an important role in mediating their biological functions [11]. One of the methods to study these interactions is fluorescence anisotropy, which is a simple, sensitive and ratio-metric measurement free from many potential artifacts [12]. The changes in anisotropy upon interactions between macromolecules using a few nanosecond fluorophore conjugate are small due to its dependence on relationship between fluorophore lifetime and rotational correlation time. A 20 ns fluorophore in comparison will significantly expand the experimental dynamic range. It should be noted that removing the autofluorescence by nanoseconds gating would also be very useful in following molecular interaction and assays either *via* fluorescence energy transfer (FRET) or *via* anisotropy in the presence of autofluorescence.

Poly-aromatics such as pyrene are among few long-lived organic fluorescent probes available. Unfortunately these probes can only be excited in UV and near-UV (typically less than 350 nm) region of the spectrum, where the autofluorescence and cellular material absorption is also strongest. The lanthanide-based probes, with their ms lifetimes, have found a role in both clinical diagnostics and in cellular imaging. Their ms lifetime allows for easy gating of all the nanosecond background. However, the intrinsic absorption of these atoms is extremely low and their use primarily depends on sensitizers with absorption in the unfavorable 300 nm–350 nm region of the spectrum. Lack of intrinsic polarization further excludes their application to study many aspects of molecular conformational motions [13]–[14]. Overall, there is need for high quantum yield long-lived small organic probes that emit in red/NIR, are photo-stable and possess high intrinsic anisotropy.

The azaoxa-triangulenium fluorophores are one such class of molecules. They belong to planar and rigid triangulenium structures, in effect a triphenyl methylium type dye bridged by either oxygen or nitrogen [15], [16]. The azaoxa-triangulenium probes, which absorb and emit in red are a sub-group of triangulenium structures in which oxygen is replaced by nitrogen and the three aromatic rings are bonded to a central carbon atom bearing a positive charge. They are similar or smaller when compared to other red fluorophores. They are characterized by a medium strong absorption. Their emissive properties, dictated by the oscillator strength of the primary transition, are unique, as the long 50 ns radiative lifetime does not result in extensive quenching of the emissive state. Consequently, the azaoxa-triangulenium dyes have high quantum yields, long fluorescence lifetimes and high limiting anisotropy [16]–[19].

In this manuscript, we characterize the fluorescence properties of two representative azaoxa-triangulenium probes ADOTA and DAOTA. We studied their photostability, fluorescence lifetimes and anisotropy when free, in solution, and conjugated to proteins. We also looked at their properties in model lipid vesicles using hexadecyl derivatives. We further used the hexadecyl-ADOTA as a membrane localized fluorescent probe and DAOTA-arginine for intra-cellular localization to carry out time gated fluorescence lifetime imaging (FLIM) of cells. Preliminary studies on time gated FLIM using ADOTA labeled secondary antibody [20], fluorescence lifetime correlation spectroscopy (FLCS) [21], and ADOTA long lifetime enabled anisotropy based immunoassay for macromolecules [22] have been recently published.

## Materials and Methods

Rabbit serum Immunoglobulin (IgG, I-5006), avidin (A-9275), streptavidin (S-4762), hexadecylamine (#445312), triethylamine (TEA, T-0886), trifluoroacetic acid (TFA, #91707), 1,1′-carbonyldiimidazole (#115533), arginine methyl ester (#11030) were purchased from Sigma-Aldrich (St. Louis MO,USA). All solvents, buffers and other common chemicals of ACS grade or better were also from Sigma-Aldrich. 1,2-dioleoyl-sn-glycero-3-phosphocholine (DOPC, 850375P) and 1,2-dimyristoyl-*sn*-glycero-3-phosphocholine (DMPC, 850345P) were from Avanti Polar Lipids (Alabaster, AL, USA). Pre-packed 5 ml Sephadex G-25 desalting columns were from Fisher Scientific (Pittsburg, PA, USA). 4-sulfo-2,3,5,6-tetrafluorophenol (STP, S-10490) was from Molecular Probes (Eugene, OR, USA). The measurements were done, unless specified, at room temperature and in either 5 mM phosphate buffer, 100 mM NaCl pH 7.4 or 5 mM Tris buffer, 100 mM NaCl pH 8.0.

### Preparation of ADOTA and DAOTA butyric acid derivatives

**Figure 1:**
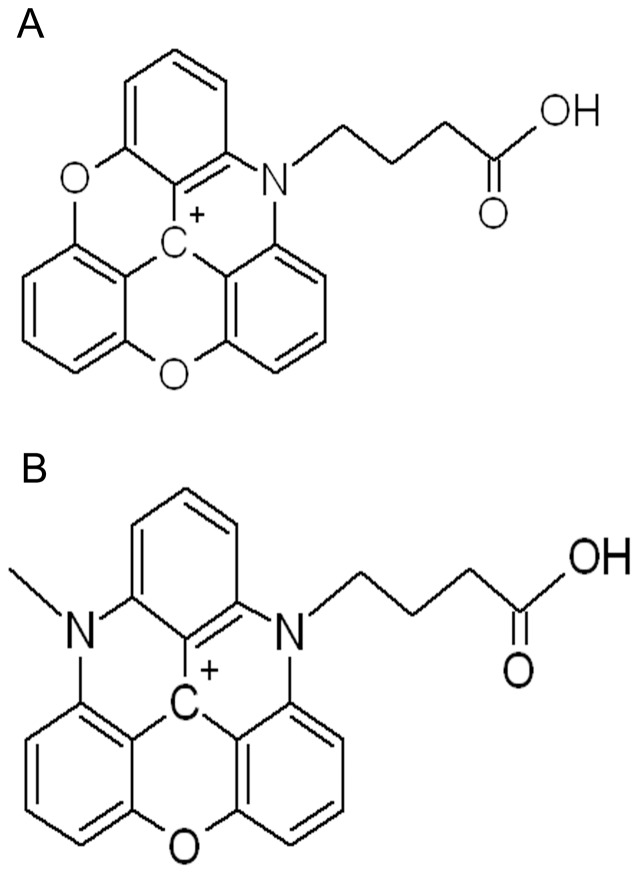
Chemical structure of ADOTA-ba. (A) and DAOTA-ba (B).

The structures of ADOTA and DAOTA butyric acid (ADOTA-ba and DAOTA-ba) are shown in Figure 1. The synthesis of ADOTA-ba is described in (20) while that of DAOTA-ba is given in supporting information (Text S1).

### Preparation of ADOTA-ba and DAOTA-ba STP ester

To 2 mg ADOTA-ba or DAOTA-ba BF_4_ salts in 350 µl dimethylformamide (DMF) were successively added 2 mol equivalents of STP and 1,1′-carbonyldiimidazole in 50 µl DMF. The reaction mixture was gently shaken overnight and resulting reactive STP esters were stored at -20°C and used as such.

### Labeling of avidin, streptavidin and IgG

Typically a small volume of reactive ester in DMF (less than 5% of final volume) was mixed with avidin, streptavidin or IgG solution in freshly prepared 100 mM bicarbonate buffer (0.5 ml to 0.8 ml) pH 8.3–8.4. The final concentrations were 3.5 µM of protein and 20 µM of reactive probe. After 4 hours of gentle shaking of the samples the protein and probe mixture was passed through a 5 ml volume Sephadex G-25 desalting column to remove the free probe. The protein molar extinctions to calculate concentrations were 105,000, 190,000 and 220,000 M^−1^ cm^−1^ respectively in case of avidin, streptavidin and IgG. Molar extinction coefficients of 12,000 and 23,000 M^−1^ cm^−1^ at 280 nm respectively for ADOTA and DAOTA were used as correction factors.

### Preparation of hexadecyl derivatives

To carry out the reaction equal volumes of DMF solution of probe active esters, hexadecylamine (3× excess) and TEA (10× excess) were mixed. The reactant mixture was protected from light and reaction allowed to take place for 24 hours with gentle shaking. The hexadecyl derivative products (ADOTA-C16 and DAOTA-C16) were separated on semi-prep (10 nm×250 mm) Kromasil C8 HPLC column. The flow rate was 2.5 ml/min. The stationary solvent was 50% acetonitrile with 0.05% TFA while the mobile gradient was a 50∶50 mixture of 2-propanol and acetonitrile containing 0.05% TFA. The hexadecyl derivatives, which eluted around 90% mobile gradient, were freeze dried and stored as DMF solution in dark at −20°C.

### Preparation of DAOTA-arginine

0.5 ml of 2 mM arginine methyl ester in freshly prepared 100 mM bicarbonate was mixed with 100 µl of 1 mM DAOTA-STP ester in DMF. The mixture was allowed to react overnight on a shaker. The DAOTA-arginine (DAOTA-Arg) was purified on semi prep (10 nm×250 mm) Kromasil C8 HPLC using a mixture of acetonitrile and 2-propanol (50∶50) containing 0.05% TFA as mobile gradient. The flow rate was 2.5 ml/min. The more polar product eluted prior (at 34% mobile phase) to reactive probe hydrolysis product DAOTA-ba (at 37% mobile phase).

### Lipid vesicle preparation

1.36 mg 1,2-dimyristoyl-*sn*-glycero-3-phosphocholine (DMPC) or 1.57 mg 1,2-dioleoyl-*sn*-glycero-3-phosphocholine (DOPC) lipids were dried from a stock chloroform solution under nitrogen and dissolved in 1 ml buffer to form multi-lamellar vesicles (MLVs) with gentle shaking. The resulting MLVs were sonicated for 5 minutes in 30-second bursts with a tip sonicator resulting in clear solution. The glass tubes with lipid solution were kept in water during sonication to avoid excess heating. The resulting small unilamellar vesicles (SUVs) were stored at 4°C after centrifugation for 5 minutes at 10000 g to remove residual MLVs and sonicator tip debris. The final lipid stock solution was 2 mM. For fluorescence measurements we added a small volume (<5 uL) of DMF solution of C16 derivative to 1 ml of 0.3 mM lipid solution in buffer to achieve lipid to probe ratio of 300. As SUVs have about 2/3 of lipid molecules in outer leaf the effective probe to lipid ratio is 200. The Lipid solution containing C16 probe was equilibrated for 3 hours at 45°C before the experiments.

### Cell cultures for FLIM

4T1 murine breast carcinoma cells from American Type Culture Collection (Manassas, VA, USA) were cultured in RPMI media supplemented with 10% FBS and 1% Penicillin-Streptomycin (Pen-Strep) solution. For FLIM experiments the cells were trypsinized using 0.5% trypsin-EDTA, centrifuged and then plated in 35 mm dishes with 10 mm glass bottom well.

### Instrumentation

The absorption spectra were recorded on Cary50 Bio UV-Visible single beam spectrophotometer (Varian, Inc) while the steady state fluorescence spectra were collected on Cary Eclipse fluorescence spectrophotometer (Varian, Inc). The sample optical densities for fluorescence measurements were typically less than 0.02 except in the case of lipid vesicles. The excitation wavelengths for steady-state measurements were respectively 506 nm and 540 nm in case of ADOTA and DAOTA unless specified. Cut-off filters OG-530 and OG-550 (Edmund Optics, Barrington, NJ, USA) were used with ADOTA and DAOTA containing lipid vesicles, respectively to eliminate scatter. To measure limiting anisotropy spectra at −40°C in frozen glycerol solution the excitation and emission wavelengths were 475 nm and 570 nm respectively in case of ADOTA-ba and 520 nm and 590 nm in case of DAOTA-ba.

The fluorescence lifetime and anisotropy decays were measured on FluoTime 200 fluorometer (Picoquant GmbH, Berlin, Germany). The fluorometer is equipped with ultrafast micro channel plate detector (MCP) from Hamamatsu, Inc. The light source was 475 nm centered laser-diode in case of ADOTA and a broader 520 nm centered light emitting diode (LED) for DAOTA. The emission wavelengths were 565 nm and 600 nm respectively in case of ADOTA and DAOTA. The instrument response function (IRF) was 60 ps full width at half maximum (FWHM) for laser diode and 500 ps in case of LED. The measurements were carried out at room temperature.

### FLIM measurements

Time-resolved images were obtained on a confocal MicroTime 200 (Picoquant GmbH, Berlin, Germany) system coupled to an Olympus IX71 microscope (Center Valley, PA). Fluorescence photons were gathered from different places of the sample using a 60× water immersion objective (N.A 1.2, Olympus). To remove scattered light, a 488 nm long-pass filter was applied. As a light source, a pulsed laser (470 nm LDH-P-C470B) with a repetition rate of 20 MHz was used. Fluorescence photons were collected with the photon-counting module (SPCM-AQR-14; PerkinElmer, Waltham, MA) and processed using the PicoHarp300 time-correlated single photon counting (TCSPC) module. Data analysis was performed using SymPhoTime (v. 5.3.2) software package (Picoquant, GmbH). In studies characterizing time gated detection cover slips with cells (4T1) were incubated with 2 ml of 5 nM of either ADOTA-C16 or DAOTA-Arg for 15 min at 37°C, 5% CO_2_ followed by image collection.

### Photostability

For photostability measurements, we prepared very thin and uniform films of ADOTA-ba, DAOTA-ba and Rhodamine B (RhB) by spin coating. Typically a drop of µM probe in 1% aqueous PVA solution was placed on a microscope cover slip and spun at 2000 rpm (Spin Coater model P6700, Specialty Coating Systems, Inc, IN, USA). The rotator first gradually accelerates and then gradually decelerates resulting in uniform and thin films. The experimental set up was same as that used for FLIM measurements.

### Life-time and anisotropy decay analysis

The intensity decays were analyzed with FluoFit v. 4 software (Picoquant, GmbH, Berlin, Germany). The lifetime intensity decays were approximated as a sum of n exponentials
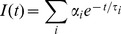
(1)


Where τ_i_ is the decay time and α_i_ the pre-exponential factor (amplitude) of the individual component (∑α_i_ = 1). The contribution of each component to the steady state intensity is given by:
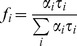
(2)


The intensity-weighted average lifetime τ_INT_ is given by:
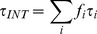
(3)


And the amplitude-weighted lifetime τ_AMP_ is given by:
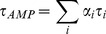
(4)


The decay of the emission anisotropy r(t) was approximated by a sum of n exponentials and associated anisotropy fraction
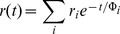
(5)and in case of lipid vesicles by an additional constant term r∞[23]–[24]:
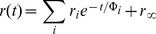
(6)


Where, Ф_i_ is rotational correlation time, r_i_ associated fraction of anisotropy and r_∞_ is residual anisotropy at long time.

The anisotropy decay parameters were determined using a nonlinear least-squares global analysis method by simultaneously fitting the vertically and horizontally polarized emission components to find a minimum set of adjustable parameters that resulted in minimum 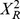 value and a random distribution of weighted residuals. The anisotropy decay analysis began between 100 ps and 150 ps in case of diode laser light source (ADOTA) and 1 ns and 1.2 ns in case of LED light source (DAOTA). Because of this tail fitting protocol we will miss anisotropy decay for those periods and the resulting recovered anisotropy will be less than limiting anisotropy.

### Rotational correlation time

For a rigid sphere rotational correlation time Ф is given by


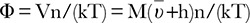
(7)

in which V is the molecular volume, n is the viscosity of the medium, k is the Boltzmann constant, T is the absolute temperature, M is the molecular weight, 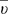 is the partial specific volume, and h is the degree of hydration. For a rigid hydrated sphere of molecular weight of 1000 Da with 0.2 g bound water per g protein, in buffer at 20°C the calculated rotational correlation time is 0.38 ns. Based on this we can expect minimum rotational correlation times for streptavidin (21 ns), avidin (26 ns) and IgG (61 ns). It should be noted that all three proteins are highly asymmetrical and that protein hydration varies between 0.2 g–0.4 g per g of protein. The asymmetry and higher bound water will lead to longer correlation time.

## Results and Discussion

The chemical structures of ADOTA-ba and DAOTA-ba are shown in Figure 1. The probes are triangular, rigid and planar with the central carbon atom formally bearing the positive charge. The charge is however significantly delocalized [16]. ADOTA-ba has one ring nitrogen while DAOTA-ba two. These nitrogen atoms are further modified to add linkers and functional groups. These probes are smaller than most probes available in this spectral region.

### Absorption spectra

**Figure 2:**
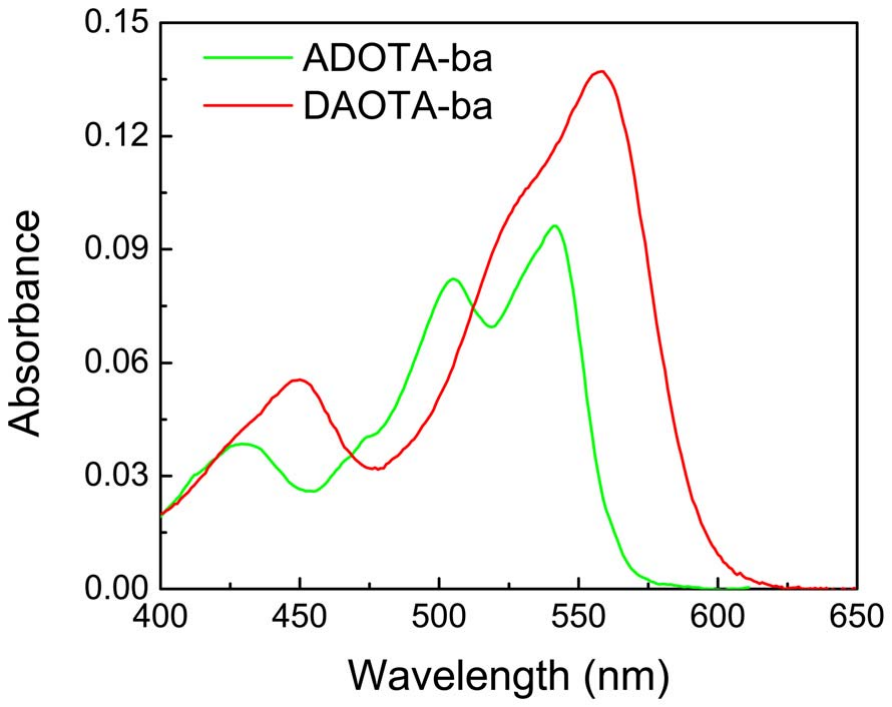
Absorption spectra of ADOTA-ba and DAOTA-ba in phosphate buffer pH 7.4.

Figure 2 shows the absorption spectra of ADOTA-ba and DAOTA-ba in phosphate buffer. In the case of ADOTA-ba the main absorption peak is at 540 nm–541 nm with a molar absorption coefficient of 9,400 M^−1^ cm^−1^. In the case of DAOTA-ba the main absorption band is located at 555 nm–556 nm while the value of the molar absorptivity is 14,000 M^−1^ cm^−1^. For both the probes in acetonitrile, we observed a minor blue shift of 1 nm while in 2-propanol a red shift of 2 nm. Further 2 nm to 3 nm red shifts were observed in ethyl acetate suggesting that the absorption spectrum is somewhat sensitive to the nature of solvent and polarity. Our observations in acetonitrile are in agreement with earlier published spectra [16]–[18]. There is also present a second transition of lower oscillator strength with peak at 448 nm in case of ADOTA and at 430 nm in case of DAOTA. With the main absorption peaks at 540 nm–541 nm (ADOTA) and 555 nm–556 nm (DAOTA) these two fluorophores can be easily excited by several widely used light sources.

**Figure 3:**
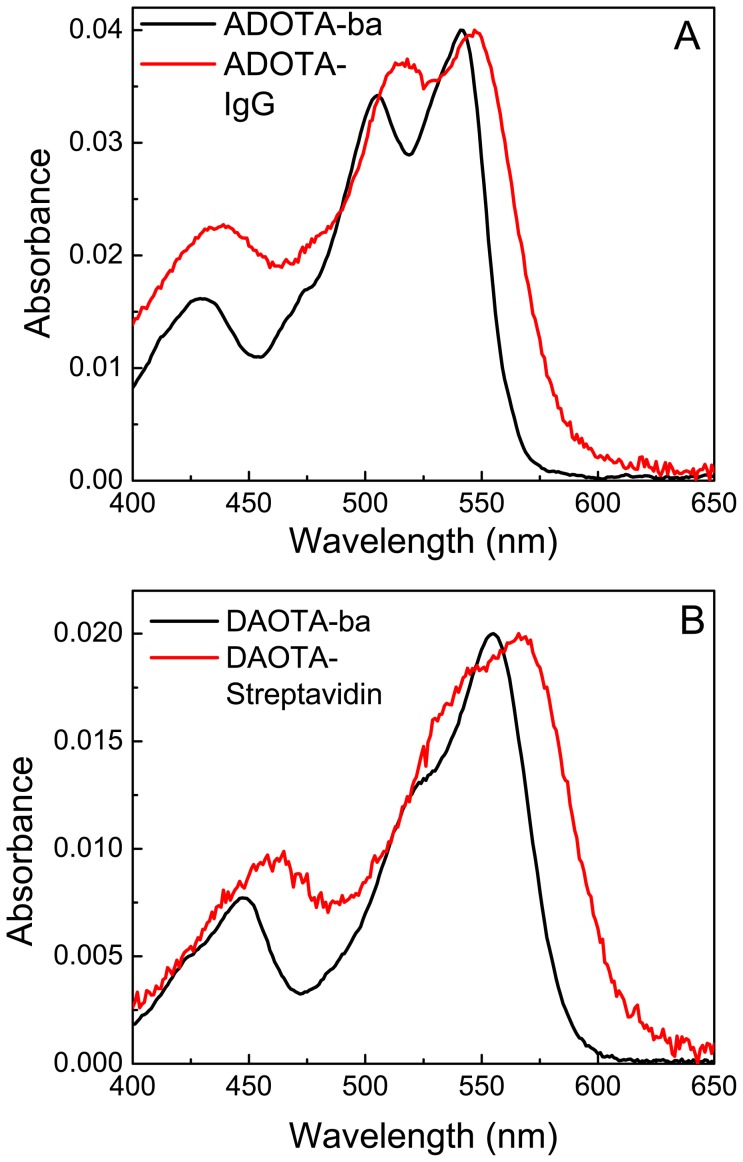
Absorption spectra of labeled proteins in Tris buffer pH 8. (A) ADOTA-ba and ADOTA-IgG (B) DAOTA-ba and DAOTA-Streptavidin.

Upon labeling to avidin, streptavidin and IgG we observed larger red-shifts in absorption spectra for both probes, when compared to their free forms in buffer solution. The shifts were between 4 nm to 10 nm. Typically, higher the labeling greater was the red shift. There was also a broadening of spectrum towards red edge for both probes. Sample spectra are shown in Figure 3A (ADOTA-IgG) and in Figure 3B (DAOTA-streptavidin). These red shifts indicate presence of interactions between the probes and the amino acid functional groups present on protein surface. Among the interacting functional groups are phenol moiety of tyrosine and indole of tryptophan (unpublished results).

### Fluorescence spectra

**Figure 4:**
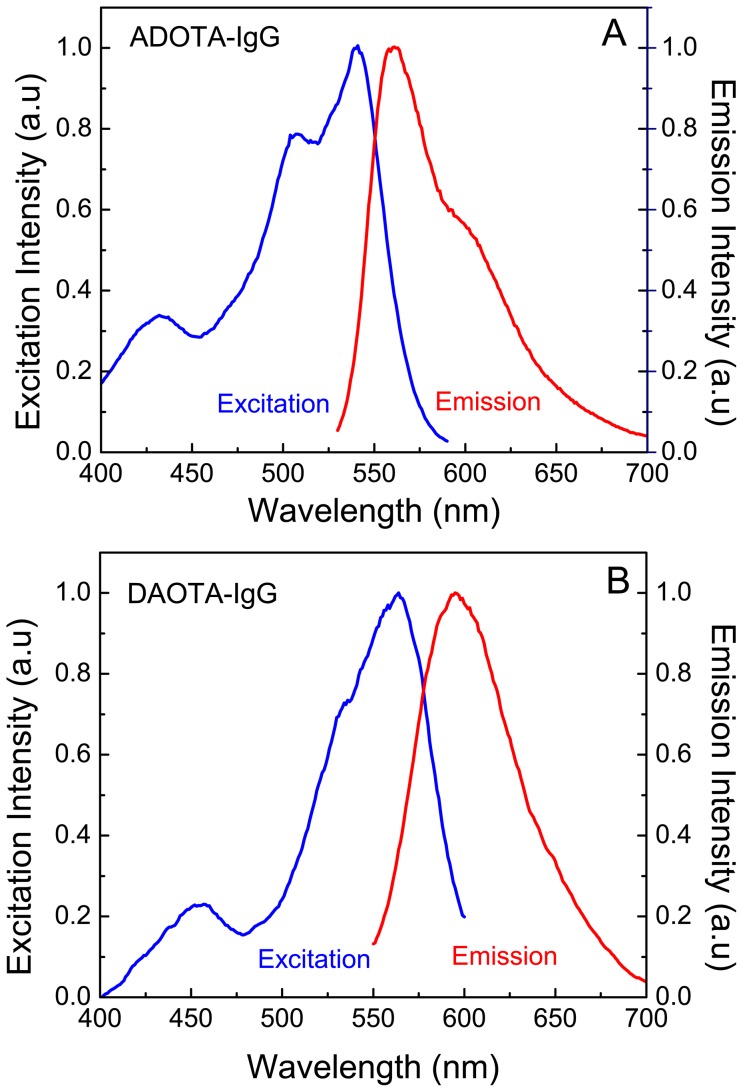
Excitation and emission spectra of labeled proteins in Tris buffer pH 8. (A) ADOTA-IgG (B) DAOTA-Streptavidin.

As expected the excitation spectra for both probes are very similar though not an exact match to their absorption spectra. The excitation spectral peaks in buffer are at 541 nm and 556 nm in case of ADOTA-ba and DAOTA-ba, respectively. The emission peaks of free fluorophores in Tris buffer are at 555 nm–556 nm and 589 nm–590 nm for ADOTA-ba and DAOTA-ba, respectively. For both fluorophores the Stokes' shifts are small resulting in significant spectral overlap between absorption and emission. Of the two probes, DAOTA has the larger Stokes' shift and lesser spectral overlap. Upon labeling to avidin, streptavidin and IgG we observed a red-shift in both excitation and emission of the probes. The excitation peaks were between 544 nm to 548 nm and the emission peaks between 560 nm to 564 nm in case of ADOTA-avidin and IgG. In case of DAOTA labeled streptavidin and IgG the respective excitation and emission wavelengths are between 560 nm to 565 nm and 593 nm to 598 nm. In general, we found that higher the degree of the labeling, the redder the spectra. As examples fluorescence excitation and emission spectra of ADOTA labeled IgG are given in Figure 4A and that of DAOTA labeled IgG in Figure 4B. The excitation fluorescence spectra are not corrected for the instrument response. Furthermore, unlike in their absorption spectrum the fluorescence populations are weighted by respective quantum yields.

### Limiting anisotropy

**Figure 5:**
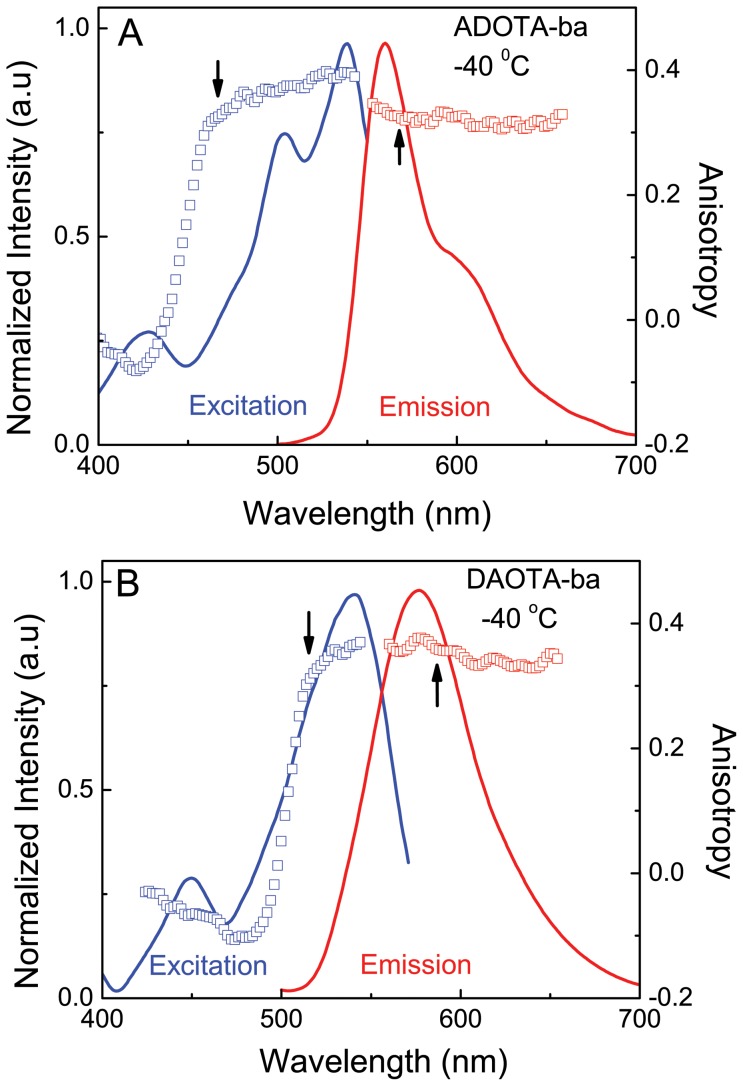
Excitation and emission intensity and anisotropy spectra in 95% glycerol at -40°C. (A) ADOTA-ba (B) DAOTA-ba. The arrows indicate wavelengths of excitation and emission.

The limiting excitation and emission anisotropy spectra were measured in 95% glycerol at -40°C, and are shown in Figure 5A (ADOTA-ba) and 5B (DAOTA-ba). The wavelengths to observe excitation and emission fluorescence and anisotropy spectra were 570 nm and 475 nm respectively in case of ADOTA. The corresponding wavelengths for DAOTA are 590 nm and 520 nm, respectively. Under these conditions, the fluorophores probes are completely immobilized when considering their fluorescence lifetimes. In case of ADOTA-ba, the limiting anisotropy has negative sign under the 430 nm peak and rises rapidly to a maximum value of 0.38 at main absorption peak. The shape of limiting anisotropy spectrum in case of DAOTA is similar to that seen with ADOTA and the maximum value of 0.36 is seen at the main absorption peak. In our instrumental set up for time-resolved anisotropy measurements, the limiting anisotropy values are 0.305 in case of ADOTA for 475 nm centered narrow diode laser and 0.326 in case of DAOTA for broader 520 nm centered LED. The observed high limiting anisotropies at absorption maxima are very similar to that seen in rhodamine and related red fluorophores [7]. Essentially similar results were obtained by Thyrhaug et al [18] for alkyl substituted ADOTA and DAOTA in frozen glycerol and PVA film. Thyrhaug et al also resolved underlying electronic transitions and their orientations in the molecular framework of these two fluorophores.

### Fluorescence lifetimes of free probes

**Figure 6:**
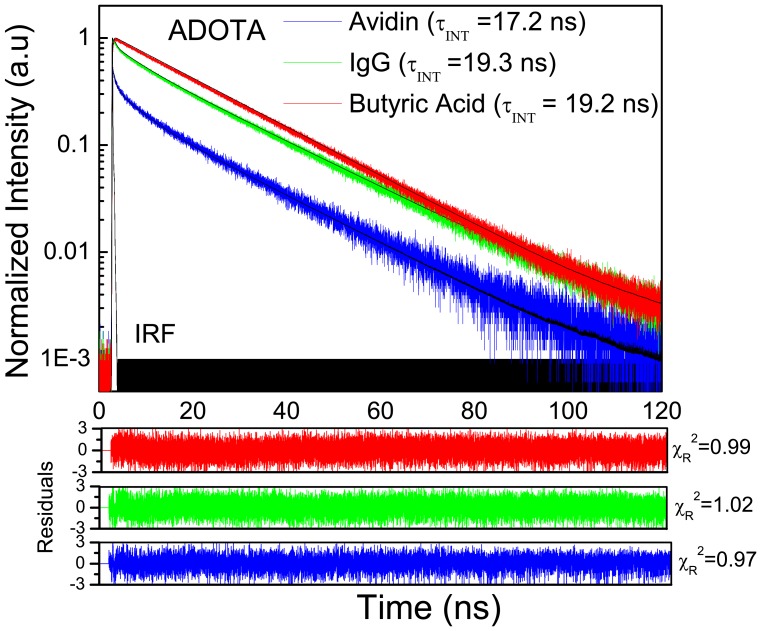
Fluorescence intensity decays of ADOTA-ba, ADOTA-IgG and ADOTA-Avidin in Tris buffer pH 8.

**Figure 7:**
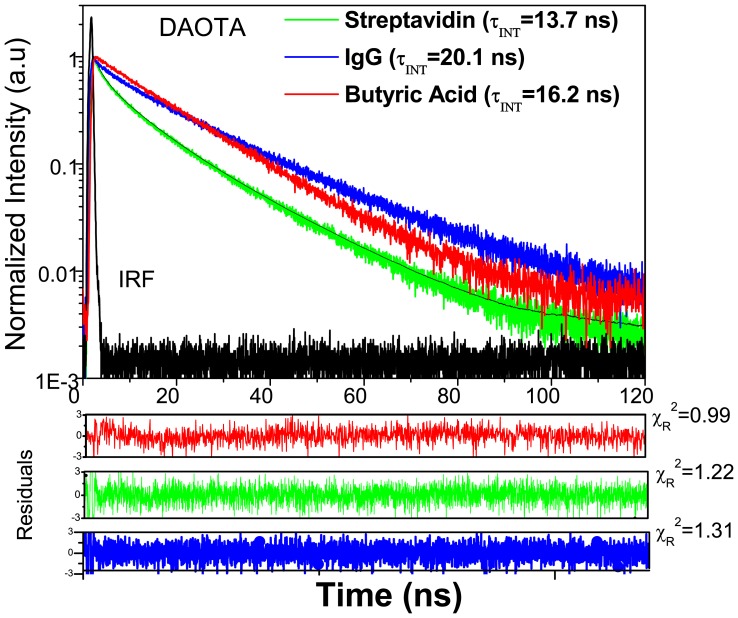
Fluorescence intensity decays of DAOTA-ba, DAOTA-IgG and DAOTA-Streptavidin in Tris buffer pH 8.

The results of fluorescence lifetime analysis of ADOTA-ba and DAOTA-ba in different solvents are presented in Table 1. The time-dependent Intensity decays of ADOTA-ba and DAOTA-ba in buffer are shown in Figures 6 and 7, respectively. ADOTA-ba in solvents mostly exhibits one lifetime between 19 ns and 22 ns. The longest fluorescence lifetime of 21.7 ns was seen in acetonitrile and the smallest of 19.2 ns in Tris buffer. The ADOTA lifetimes in propylene glycol (PG) and triacetin were 20.8 ns and 20.9 ns, respectively. In 95% glycerol, the decay is heterogeneous with the presence of a small population of a shorter lifetime component (Table 1).

**Table 1:** Fluorescence lifetimes of ADOTA-ba and DAOTA-ba in solvents.

Sample	Lifetime (ns)		Amplitudes		Average Lifetime (ns)		X^2^_R_
	τ_1_	τ_2_	α_1_	α_2_	τ_AMP_	τ_INT_	
			**ADOTA-ba**				
Acetonitrile		21.65		1	21.65	21.65	0.949
Buffer		19.18		1	19.10	19.10	0.995
Triacetin		20.90		1	20.90	20.90	1.007
PG		20.82		1	20.82	20.82	1.045
Glycerol	1.25	19.84	0.138	0.862	17.28	19.55	1.007
			**DAOTA-ba**				
Acetonitrile		18.95		1.00	18.95	18.95	1.064
Buffer		16.23		1.00	16.23	16.23	0.993
Triacetin		19.95		1.00	19.95	19.95	1.665
Glycerol	2.98	17.31	0.077	0.923	16.20	17.11	0.982

* Both acetonitrile and PG contain 1% while the glycerol 5% by volume 1 mM tris buffer.

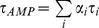
, χR2 = Goodness of fit.

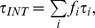
Where 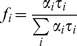

In general, DAOTA-ba fluorescence lifetimes were shorter than those seen for ADOTA-ba in each solvent (Table 1). This is not surprising as DAOTA is both red-shifted and has higher molar absorption coefficient. The measured lifetimes are 19 ns in acetonitrile, 19.9 ns in triacetin and 16.2 ns in buffer. The decay in 95% glycerol is again heterogeneous with a major lifetime component of 17.3 ns. Our values in acetonitrile are somewhat lower than earlier reports, some of them in oxygen free solvent [16]–[18]. Another difference is presence of small amount of buffer (1% in acetonitrile and 5% in glycerol) in present study. Overall, these are among the longest reported lifetimes for red emitting simple organic fluorophores. For a comparison, the measured lifetimes for spectrally similar probes are typically less than 4 ns [7].

### Lifetimes of labeled proteins

The results of multi-component lifetime analysis of the fluorescence intensity decays of ADOTA labeled IgG and avidin and DAOTA labeled IgG and streptavidin are given in Table 2. The intensity decays for the conjugated probes are heterogeneous and require three lifetime components. We found a highly quenched, a somewhat quenched and a nearly unquenched population. These three populations have respectively sub-nanosecond to nanosecond, 5 ns to 10 ns and 18 ns to 23 ns lifetimes. In general, we observed relatively more of highly quenched population and less of unquenched population in avidin and streptavidin when compared to IgG. The trend is also reflected in amplitude averaged ADOTA life times of 13.3 ns and 9.5 ns respectively for IgG and avidin. In the case of DAOTA, we have respective amplitude averaged lifetimes of 16 ns and 8.2 ns. It should be noted that IRF FWHM in case of LED based DAOTA measurements is 0.5 ns when compared to 60 ps for diode laser light source used for ADOTA measurements. An IRF FWHM which is comparable or bigger than sub-nanosecond lifetime component will lead to significant underestimation of the highly quenched DAOTA population and an apparent higher amplitude averaged lifetime. For comparison in buffer the fluorescence lifetime is 19.2 ns in case of ADOTA-ba and that is 16.2 ns for DAOTA-ba.

**Table 2:** Fluorescence lifetimes of ADOTA and DAOTA labeled IgG, Avidin and Streptavidin.

Sample	Lifetime (ns)			Amplitudes			Average Lifetime (ns)		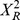
	τ_1_	τ_2_	τ_3_	α_1_	α_2_	α_3_	τ_AMP_	τ_INT_	
				**ADOTA**					
IgG	0.63	8.35	21.17	0.256	0.204	0.540	13.30	19.27	1.02
Avidin	0.55	5.00	19.34	0.344	0.236	0.420	9.49	17.18	0.977
				**DAOTA**					
IgG	1.99	9.62	22.67	0.145	0.282	0.573	16.01	20.11	1.313
Streptavidin	1.63	7.26	18.20	0.385	0.335	0.280	8.16	13.67	1.227

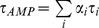
, χR2 = Goodness of fit.

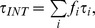
Where 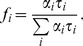

The fluorescence intensity decays for ADOTA and DAOTA labeled proteins are presented in Figures 5 and 6, respectively. We also included the decays of the free probe in the Figures which shows both the heterogeneity and the presence of highly quenched population at very early times in the decays of labeled proteins. Also given in the figures are intensity-averaged lifetimes. These lifetimes represent decay characteristics of emitted photons during an experiment. The intensity-weighted lifetimes of most of labeled probes are close to those seen for free probe and range between 14 ns and 20 ns. The quenching of organic fluorescent probes up on protein conjugation is a frequent occurrence. Typically, the susceptibility to quenching decreases as fluorophore spectrum shifts towards far red and NIR. Among the quenchers are functional groups from amino acids tryptophan, tyrosine, histidine and methionine [25]–[27]. Electron transfer has been shown as one of the major quenching mechanisms [28]. Azaoxatriangulenium fluorophores are also quenched by photo-induced electron transfer [17], [18], [29]. ADOTA acts as an electron acceptor while DAOTA can both donate and accept an electron. One of the practical remedy to minimize protein surface mediated quenching of fluorophores is to increase the length of the linker.

### Anisotropy decays of free probes

**Figure 8:**
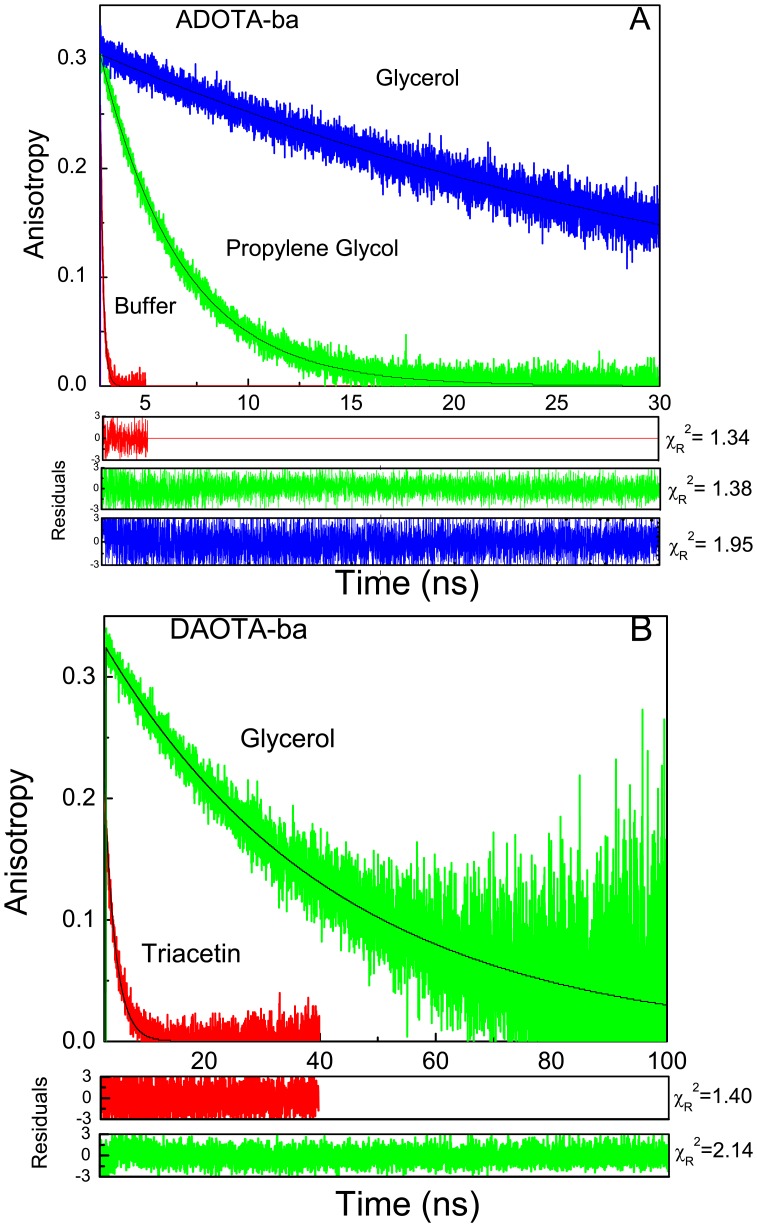
Anisotropy decays of ADOTA and DAOTA at room temperature. (A) ADOTA-ba (B). DAOTA-ba.

The anisotropy decays of ADOTA-ba in tris buffer, PG and 95% glycerol are shown in Figure8A and those for DAOTA-ba in triacetin and 95% glycerol in Figure 8B. As expected the very fast motions in buffer results in extremely rapid decay of anisotropy, while significantly slower motions in more viscous media can be seen as very slow decline of anisotropy with time. Same trend is visible between triacetin and 95% glycerol in case of DAOTA-ba. The measured rotational correlation times and associated anisotropy at room temperature are given in Table 3 for both the probes. It should be noted that our anisotropy decay analysis using tail fitting results in time gating of initial 100 ps to150 ps decay in case of diode laser (ADOTA-ba) and 1 ns to 1.2 ns (DAOTA-ba) in case of LED light source. The magnitude of missing fraction of total anisotropy will depend up on rotational correlation time values. If the time gate and rotational correlation times are comparable, we will be unable to recover a significant part of total anisotropy. On the other end when the rotational correlation time is >> the time gate we will recover almost all of the limiting anisotropy. As expected the recovered correlation times follow the bulk solvent viscosity which is expected to increase in the order acetonitrile < buffer << triacetin < PG << 95% glycerol. For ADOTA-ba in acetonitrile even after missing a significant part of anisotropy decay, we were able to recover a rotational correlation time of around 100 ps. The value is reasonable for a solvated ellipsoid of molecular weight (MW) of 370 Da. However, as expected from missing initial 100 ps of anisotropy decay the recovered anisotropy of 0.171 is significantly lower than the expected limiting anisotropy of 0.305. The correlation time for ADOTA-ba in buffer is 150 ps and as expected somewhat higher recovered anisotropy value of 0.268. The rotational correlation times are respectively 2.5 ns, 4 ns and 38 ns in more viscous triacetin, PG and 95% glycerol and follow the solvent bulk viscosity. With values of Ф >>100 ps–150 ps time gate the recovered associated anisotropy of 0.304–0.305 is now comparable to the limiting anisotropy measured in frozen glycerol at −40°C.

**Table 3:** Anisotropy decays of ADOTA-ba and DAOTA-ba in solvents.

Sample	ADOTA-ba			DAOTA-ba		
	Φ (ns)	r	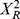	Φ (ns)	r	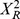
Acetonitrile	101±6 ps	0.171	1.241	ND		
Buffer	147±5	0.268	1.345	ND		
Triacetin	2.5±0.1	0.304	1.490	1.9±0.1	0.275	1.407
PG	4.0±0.1	0.305	1.383	ND		
Glycerol	37.7±0.9	0.305	1.959	40.7±1	0.328	2.144

* Both acetonitrile and PG contain 1% while the glycerol 5% by volume 1 mM tris buffer.

ND: not determined.

The DAOTA-ba rotational correlation times were 1.9 ns and 40 ns in case of triacetin and 95% glycerol, respectively. The measured anisotropy values were 0.275 and 0.328, respectively. As expected the measured anisotropy from the decay in 95% glycerol is same as that seen in frozen glycerol from steady state measurement due to Ф >>1 ns time gate. The rotational correlation times of both DAOTA-ba and ADOTA-ba in triacetin and 95% glycerol are comparable as expected for molecules of similar size. The minor differences are most likely due to day-to-day variations in room temperature, as the measurements were done without use of a temperature control device.

### Anisotropy decays of labeled avidin, streptavidin and IgG

**Figure 9:**
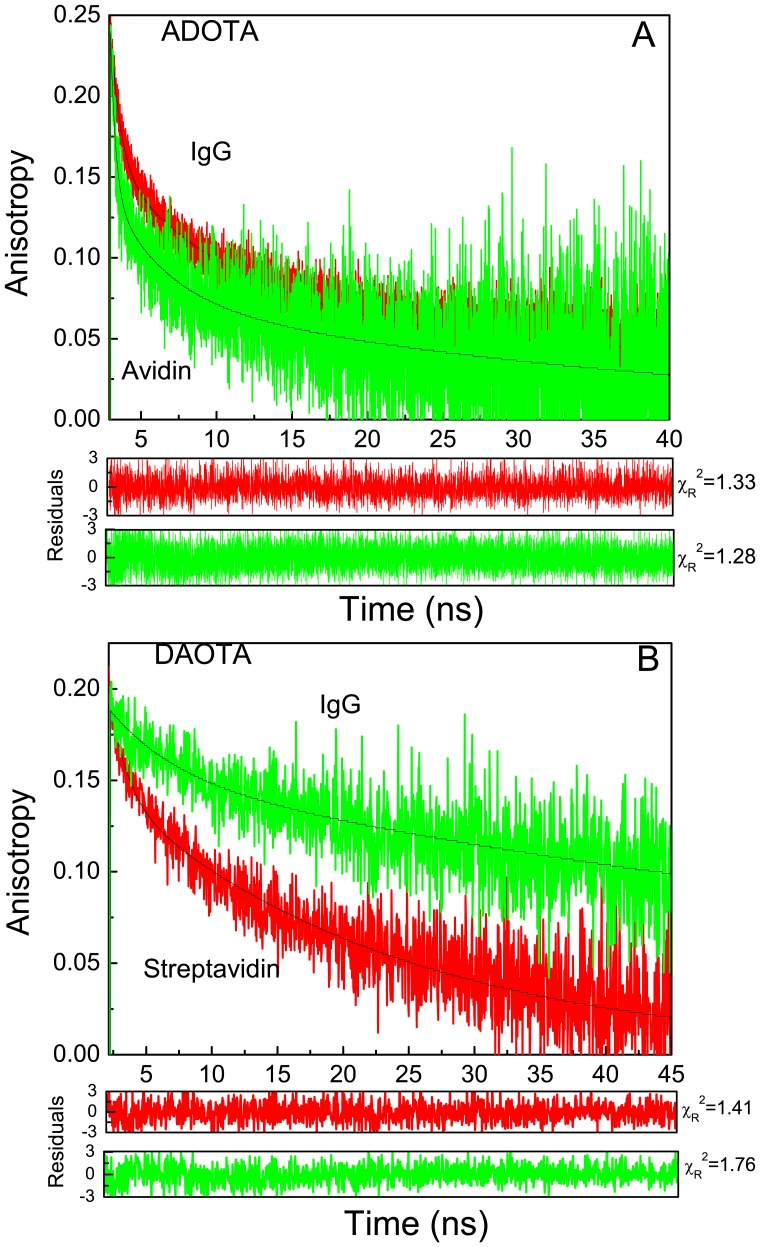
Fluorescence anisotropy decays of labeled proteins. (A) ADOTA Avidin and ADOTA-IgG (B) DAOTA-Streptavidin and DAOTA-IgG.

The results from analysis of anisotropy decays of labeled proteins are given in Table 4. The anisotropy decays are shown in Figure 9A and 9B for ADOTA labeled IgG and avidin and DAOTA labeled streptavidin and IgG, respectively. The anisotropy decays are heterogeneous, with presence of up to three correlation times. The sub-nanosecond to nanosecond correlation time represents local motional freedom of the labeled probe. Somewhat slower intermediate motions of 3 ns to 6 ns correlation times are likely due to segmental motions of secondary structure elements. The longest correlation times reflect over all tumbling of the protein macromolecules. The correlation times of 80 ns to 85 ns in case of IgG (MW 150 kDa to 160 kDa), 25 ns in case of streptavidin (MW 54 kDa) and 37 ns for avidin (MW 64 kDa) are reasonable for respective asymmetrical globular proteins. The anisotropy fraction associated with rotational diffusion of proteins in case of DAOTA is between 0.129 and 0.158 when compared to the value of 0.066 to 0.076 with ADOTA. This may reflect more hydrophobic character of DAOTA and enhanced propensity to stick to protein surface when compared with ADOTA.

**Table 4:** Anisotropy decays of ADOTA and DAOTA labeled avidin, streptavidin and IgG.

Sample	Correlation Times (ns)			Amplitudes			<r>	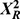
	Φ_1_	Φ_2_	Φ_3_	r_1_	r_2_	r_3_		
			**ADOTA**					
Avidin	0.4±0.1	3.5±0.9	36.6±7.4	0.100	0.064	0.076	0.239	1.284
IgG	0.65±0.1	5.7±1	80.1±12.7	0.096	0.093	0.066	0.255	1.330
			**DAOTA**					
Streptavidin	-	2.7±0.5	25.2.±1.7	-	0.054	0.129	0.183	1.418
IgG	-	3.4±1.4	84.7±8.3	-	0.033	0.158	0.191	1.769

<r> is expected to be 0.305 and 0.325 for ADOTA and DAOTA respectively at zero time. Due to tail fitting procedure the analysis of the anisotropy decays start from 150 ps in case of ADOTA and 1 ns in case of DAOTA.

The recovered anisotropy in case of labeled avidin, streptavidin and IgG is significantly lower than expected limiting anisotropy of 0.305 in case of ADOTA and 0.325 in case of DAOTA. We believe main reason is the presence of sub-nanosecond motions that we partially miss in tail fitting procedure. The results also suggest that in present form, most likely due to the presence of three flexible methylene groups in the butyric acid linker, both probes when labeled to a protein will have considerable motional freedom. The resolution of complex motions from anisotropy decay is interplay between the relative values of rotational correlation times, associated fractions of anisotropy and the fluorophore lifetime. It is therefore preferable that fluorescent probes are less flexible with more of anisotropy associating with tumbling of whole protein. Derivatives of ADOTA and DAOTA, with less flexibility will increase the capability to resolve complex motions and longer correlation times. Our results suggest that with help of long fluorescence lifetime probes of high intrinsic anisotropy, it is possible to both resolve the intermediate intra-molecular motions and measure over all rotational diffusion even when associated anisotropy fraction is rather small. For example, the value of anisotropy associated with global motions is only 0.066–0.076 in case of ADOTA out of total anisotropy of 0.306. It should also be obvious that a 3 ns lifetime fluorophore will not be able to reliably measure very large correlation times, as fluorescence signal would have ended very early during the tumbling of the molecule. A typical noise free anisotropy decay observation window for 3 ns fluorophore is less than 15 ns.

### ADOTA and DAOTA in lipid vesicles

**Figure 10:**
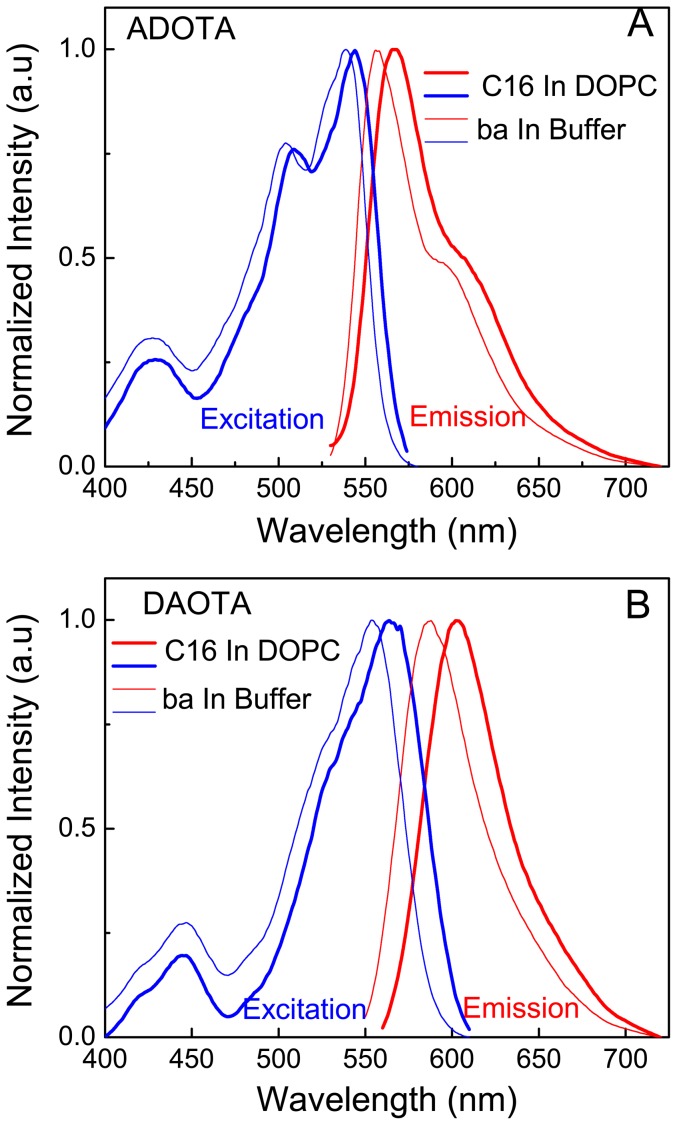
Fluorescence excitation and emission spectra of hexadecyl derivatives in DOPC. (A) ADOTA (B) DAOTA.

Figures 10A and 10B show excitation and emission spectra of ADOTA and DAOTA hexadecyl derivatives in DOPC small vesicles. The probe concentrations were one probe for every 200 lipid molecules. The excitation and emission spectra for both ADOTA and DAOTA are red-shifted in the DOPC lipid vesicles, when compared to free butyric acid derivatives in phosphate buffer. The excitation and emission peaks for ADOTA in DOPC are at 545 nm–546 nm and 566 nm–567 nm representing red shifts of 5 nm in excitation and 11 nm in the emission spectrum. In the case of DAOTA excitation and emission peaks of 566 nm and 602 nm–603 nm represent red-shifts of 9 nm and 12 nm, when moving from an aqueous environment to the phospholipid head group/interface region. We did not characterize the probe location in the lipid vesicle. Due to the presence of a positive charge in the planar azaoxa-triangulenium molecules, the probes are unlike to penetrate into the lipid hydrocarbon core and are most likely present in strongly dipolar head group/interface region. As these red shifts are significantly larger than seen in 2-propanol, there are other factors involved beside simple polarity of the microenvironment. The local oriented dipoles of DOPC and counter ion effects are most likely the origin of these spectral shifts [30]–[31].

**Figure 11:**
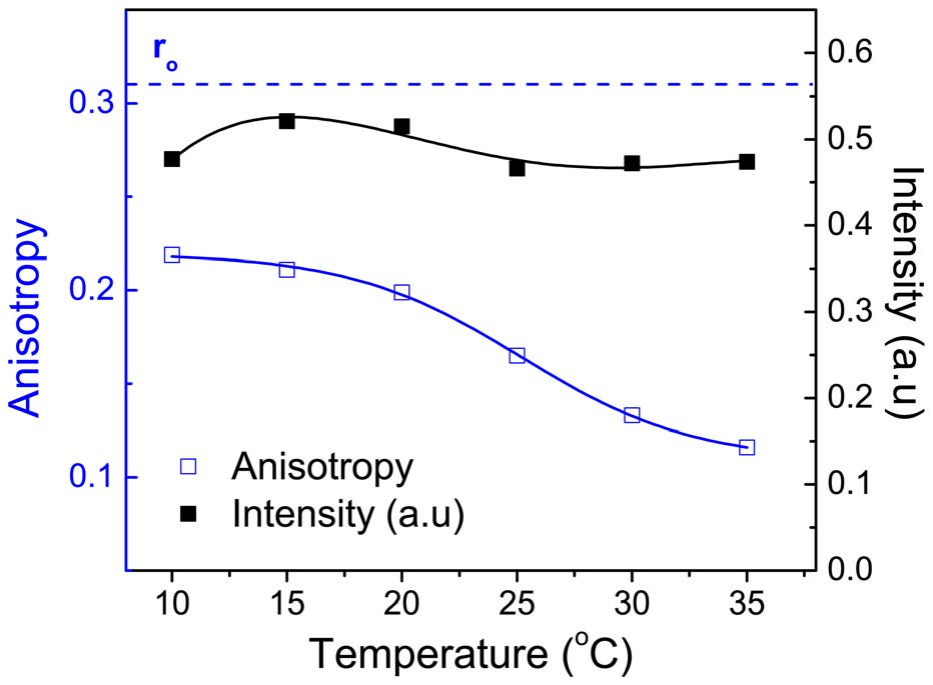
Fluorescence intensity and steady state anisotropy of ADOTA-C16 in DMPC between 10°C and 35°C.

We also looked at the steady-state anisotropy and fluorescence emission of hexadecyl-ADOTA in DMPC small vesicle during the transition from gel to liquid crystalline state. The results are shown in Figure 11. The fluorescence intensity decreases by less than 5% between 15°C and 35°C. Even though it is possible to see the subtle signature of phase transition, the change in emission maxima is rather small (less than 4 nm). There is also a reproducible small decrease in the intensity between 15°C and 10°C. We suspect it is primarily due to inner filter effect as the vesicles become more opaque up on lowering of temperature. The steady-state anisotropy decreased from 0.219 at 10°C to 0.114 at 35°C, reflecting the phase transition of DMPC. Our results above phase transition are similar to those seen with anchored DPH derivatives [23, 24, and 32] and fluorescent fatty acids with long lifetime [33], [34]. It should be noted that the fluorophore in DPH derivatives and fluorescent fatty acids are present in the hydrocarbon region of lipid while the ADOTA and DAOTA moieties are more likely to be at interface/head-group region, with the hexadecyl moiety aligning with the hydrocarbon core of the lipid.

**Figure 12:**
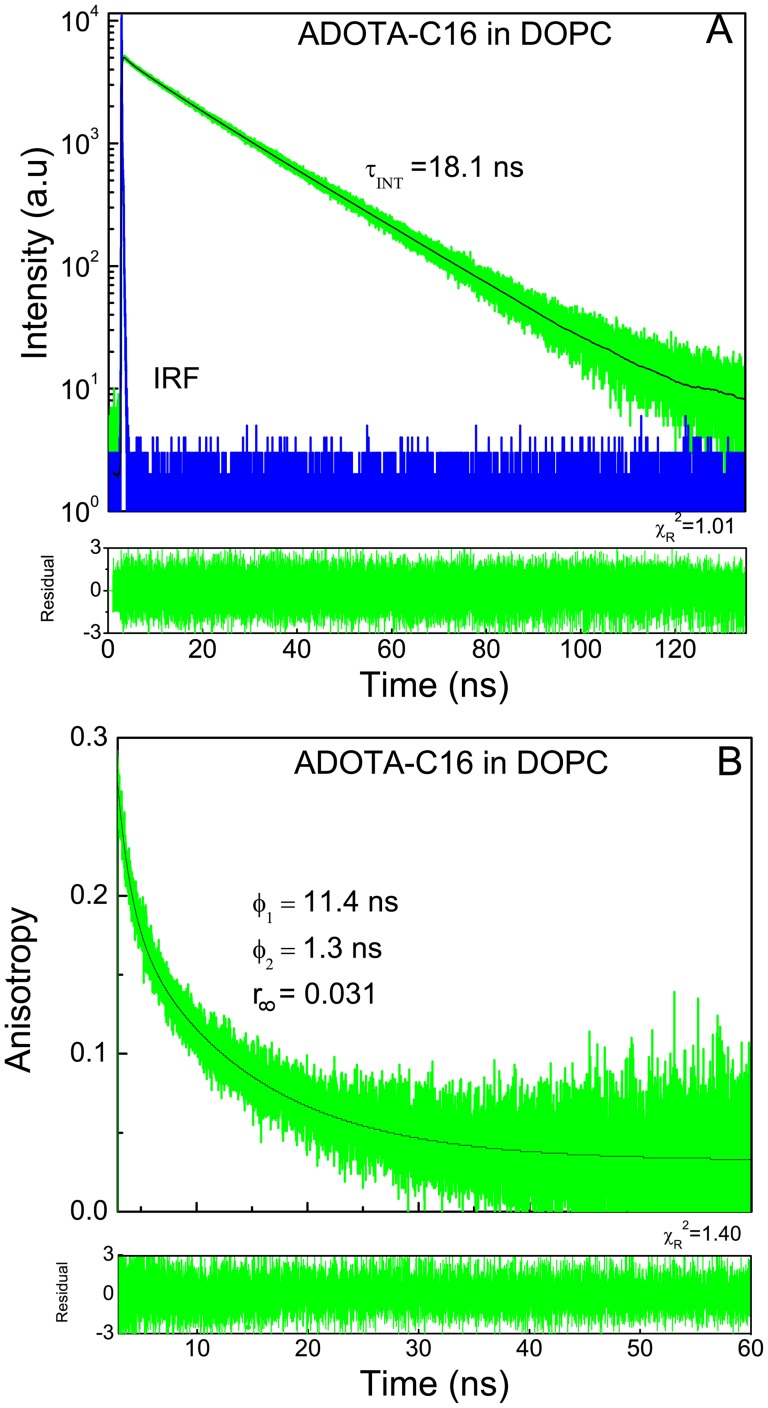
ADOTA-C16 in DOPC. (A) Fluorescence intensity decay (B) anisotropy decay.

**Figure 13:**
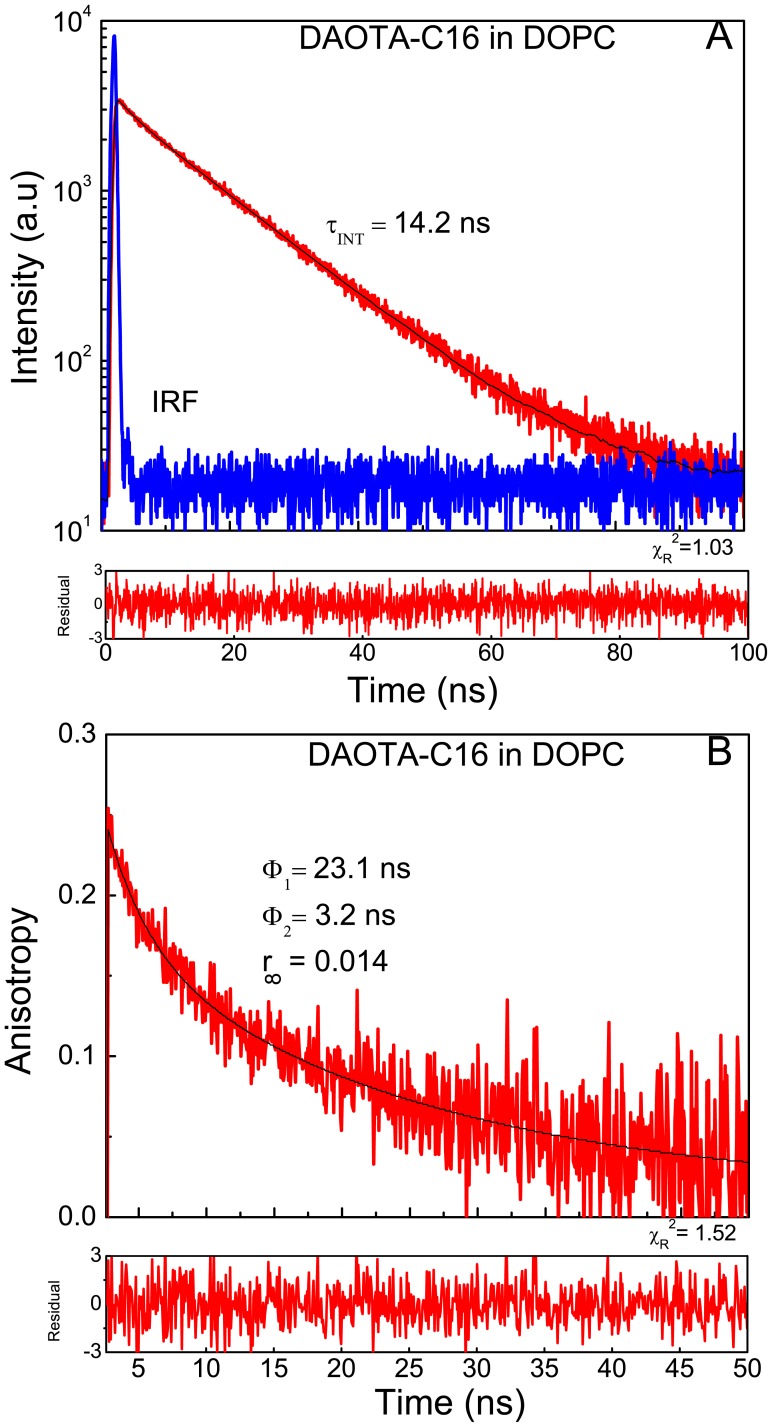
DAOTA-C16 in DOPC. (A) Fluorescence intensity decay B anisotropy decay.

The results from fluorescence lifetime measurements in DOPC for both ADOTA and DAOTA hexadecyl derivatives are given in Table 5. The intensity and anisotropy decays for ADOA-C16 are shown in Figures 12A and 12B and of DAOTA-C16 in Figures 13A and 13B. The lifetime of main component was 18.5 ns in case of ADOTA-C16 and 14.6 ns for DAOTA C16 derivative. There is also a small population with a short lifetime, suggesting some heterogeneity of the environment. The heterogeneity in the intensity decays when in lipid environment in otherwise single lifetime probe has been observed with several fluorophores. Among possible explanations are presence of micro-heterogeneity in vesicles and less than random distribution of the fluorophore [33], [35]. Table 6 has results from anisotropy decays in DOPC. We can satisfactorily describe the anisotropy decays with two correlation times and a residual anisotropy often known as r_∞_. As expected in case of a disordered lipid DOPC the values of r_∞_, which reflects “order”, is small. It is less than 10% of total anisotropy in case of ADOTA-C16 and even less around 5% in case of DAOTA-C16. The recovered anisotropy of 0.278 and 0.246 for ADOTA and DAOTA respectively are smaller than their limiting anisotropy values due to tail fitting of anisotropy decays in the presence of nanosecond probe motions. We are time gating initial 150 ps and 1 ns in case of ADOTA-C16 and DAOTA-C16, respectively. If we were to keep this under-estimation of faster component in mind, the probes would seem to experience a faster and almost order of magnitude slower motions of equal amplitudes. It should be noted that our model of sum of correlation times and a residual anisotropy analysis [23] is often applied due to its simplicity. Models that are more physical are available which also tend to be more complex [24, 32, 34, and 35]. These results also compare favorably to those seen with long lifetime fluorescent fatty acids [33], [34]. The fluorescence lifetimes of these fatty acids are similar to the azaoxa-triangulenium fluorophores. However, though anchored in head-group/interface region by their carboxylic group the fluorescent part of the fatty acid is located in hydrocarbon core.

**Table 5:** Fluorescence lifetimes of ADOTA-C16 and DAOTA-C16 in DOPC Vesicles.

Sample	Lifetime (ns)		Amplitudes		Average Lifetime (ns)		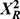
	τ_1_	τ_2_	α _1_	α _2_	τ_AMP_	τ_INT_	
ADOTA-C16	5.94	18.75	0.148	0.852	16.85	18.08	1.014
DAOTA-C16	2.51	14.55	0.134	0.866	12.93	14.23	1.027

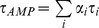
, χR2 = Goodness of fit

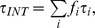
Where 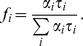

**Table 6:** Anisotropy decays of ADOTA-C16 and DAOTA-C16 in DOPC Vesicles.

Sample	Correlation Times(ns)			Amplitude		<r>	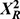
	Φ_1_	Φ_2_	r_1_	r_2_	r_∞_		
ADOTA-C16	1.3(.2)	11.4(.5)	0.094	0.153	0.031	0.278	1.44
DAOTA-C16	3.2(.4)	23.1(1.4)	0.077	0.155	0.014	0.246	1.518

### Photostability

**Figure 14:**
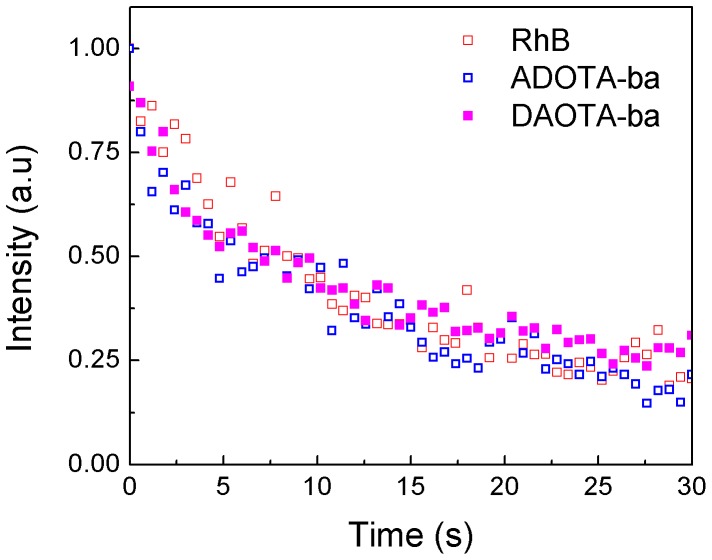
Photostability of RhB, ADOTA-ba and DAOTA-ba.

As shown in Figure 14 the photostability of both ADOTA and DAOTA is comparable to that of RhB. As fluorescence lifetime is one of the factors that determine the photostability, these results suggest that both azaoxatriangulenium probes are in reality several-fold less reactive in their excited state than RhB as their lifetimes are at least 7 times longer.

### Fluorescence lifetime imaging (FLIM)

**Figure 15:**
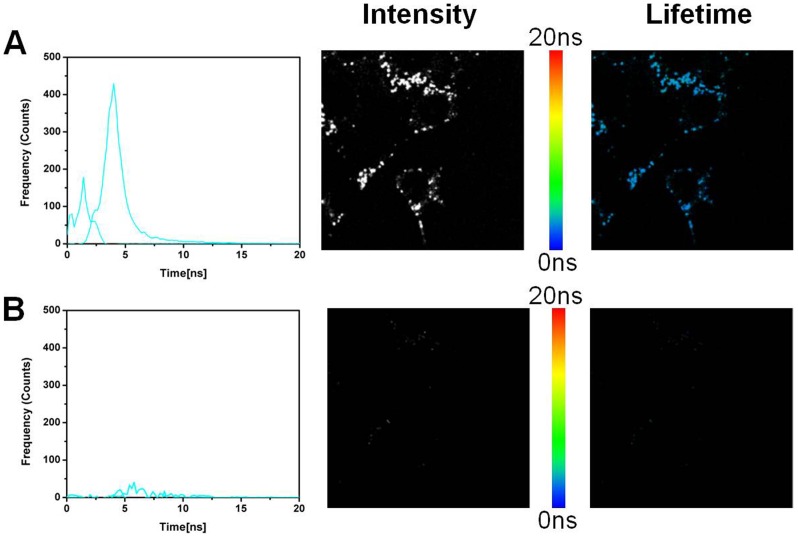
Fluorescence lifetime imaging (FLIM) of the 4T1 endothelial cell line. (A) Cell autofluorescence image. (B) Cell autofluorescence image after time gating.

**Figure 16:**
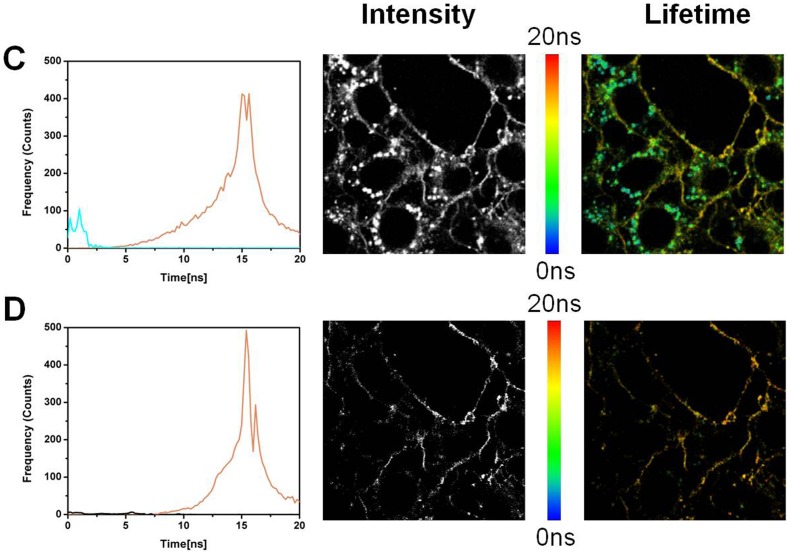
Fluorescence lifetime imaging (FLIM) of the 4T1 endothelial cell line stained with ADOTA-C16. (A) ADOTA-C16 stained 4T1 cell line (B) ADOTA-C16 stained 4T1 cell line after time gating.

**Figure 17:**
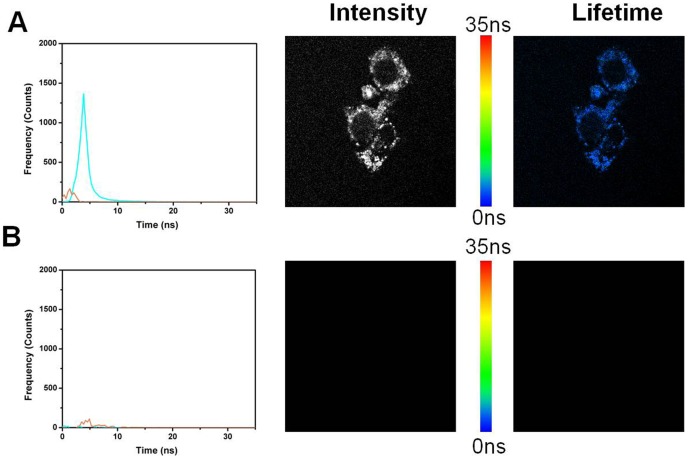
Fluorescence lifetime imaging (FLIM) of the 4T1 endothelial cell line. (A) Autofluorescence image (B) Autofluorescence image after time gating.

**Figure 18:**
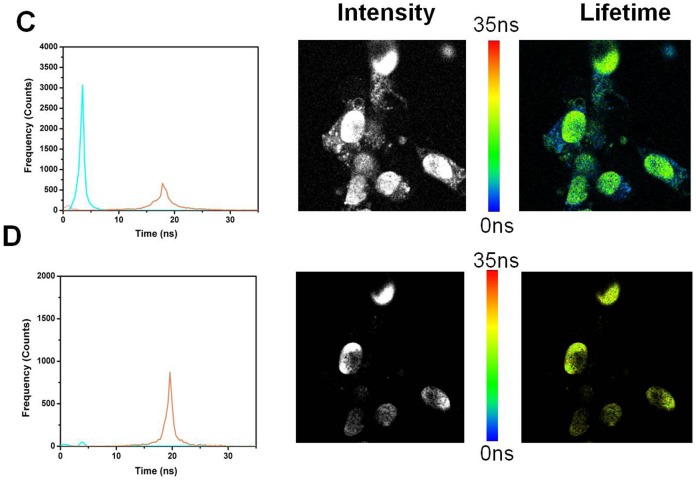
Fluorescence lifetime imaging (FLIM) of the 4T1 endothelial cell line stained with DAOTA-Arg. (A) DAOTA-Arg stained 4T1 cells (B) DAOTA-Arg stained 4T1 cells after time gating.

The results with ADOTA-C16 are shown in Figures 15 A–B and Figure 16 A–B, while that of DAOTA-Arg are in Figure 17A–B and 18 A–B. A breast cancer endothelial cell line 4T1 was used to collect these images. All four panes in the Figures 15, 16, 17, and 18 are made up of three parts. The left most panel shows photon counts and associated fluorescence lifetimes for cell autofluorescence. The middle part is an intensity image and the right most is a lifetime image of same cells. The auto-fluorescence signal from the cells can be satisfactorily described by two lifetimes of 1.3 ns and 3.2 ns. We can see that after 10 ns almost all of the fluorescence has disappeared (Figure 15A). We therefore used a gate of 10 ns and discarded all the photons collected before this point to construct time gated FLIM images.The results of this 10 ns time gating are shown in panel B of the figure 15. The left most image has few residual photons as result of the near complete elimination of autofluorescence signal with the time gating. The intensity (middle) and the lifetime (right most) images are now essentially black. Figure 16 panels A and B depict the FLIM images collected from cells that were stained with 5 nM ADOTA-C16 solution. We now observe presence of an additional relatively long-lived component of 15.6 ns, which also is the dominant source of photons (Pane A). As expected the middle image is bright with fluorescence signal covering significant parts of cell surface. As this long-lived component affects the average lifetimes calculated for each pixel it results in making the false color in the lifetime image appear more yellow/red (right most figure). When time gating is applied as before, the two short-lived components attributed to auto-fluorescence are eliminated leaving only the long-lived ADOTA signal (Pane B left most panel). The intensity is significantly reduced in middle panel and fluorescence is now confined to cell membrane area. The color of the FLIM image in right most part is now noticeably redder, as the blue was removed with the time gating. It should be emphasized that 10 ns time gating not only eliminates auto-fluorescence but also the initial part of ADOTA fluorescence decay. The result is dimmer fluorescence images. However, we also obtain a significantly improved signal contrast when compared to non-gated images (right most panels A vs B)

The FLIM results for DAOTA-Arg (Figure 17 A–B and 18 A–B) labeled cells are organized similar to those seen with ADOTA-C16 in Figure 15 and 16. Again we can see presence of autofluorescence of nanosecond lifetimes in leftmost panel of 17 A and 18A and its elimination with a 10 ns time gate in leftmost B panels. The lifetime histogram for DAOTA-Arg is centered on 18 ns–19 ns (Figure 17 A left most panel). The results from time gated intensity and lifetime FLIM are given in middle and right most panels of B Pane of Figures 17 and 18. The resulting images though weaker in over-all signal have again considerably improved signal to noise and contrast because of the gating.

## Conclusion

We find that the red emitting azoxa-triangulenium probes ADOTA and DAOTA are bright, reasonably photostable, have fluorescence lifetimes approaching 20 ns when free and possess high intrinsic anisotropy. They are also not very sensitive to solvent polarity as we observe only a minor red shift when going from aqueous environment to 2-propanol. They are comparable or smaller than most commonly used red fluorophores. Upon labeling to avidin, streptavidin and IgG we observed moderate red-shift in their absorption and emission and presence of significant quenched population. The quenching of red and NIR organic probes at protein surface is a known phenomenon. Among the quenchers are indole moiety of tryptophan and phenol of tyrosine amino acids [25]–[27]. The quenching processes involve photo-induced electron transfer [28]. Studies are in progress to systematically characterize the protein surface groups mediated quenching processes. In addition, to minimize these quenching interactions we are exploring different linkers as well as the lengthening of linker, which has proven to minimize such interactions and increase the quantum yields of protein labeled probes [7]. As expected from relatively long lifetime we were able to measure rotational correlation time of large macromolecules like avidin, streptavidin and IgG. For obvious reasons this cannot be done with fluorescence probes with lifetimes of few nanoseconds. We were also able to observe both local probe flexibility and somewhat slower internal protein motions. It was even more remarkable as labeled probes turned out to quite flexible and effectively reduced the anisotropy associated with both intermediate and global motions. It should be obvious that a more rigidly labeled ADOTA or DAOTA will provide greater dynamic range and better resolution. Our preliminary characterization of the probes in lipid environment indicates that both ADOTA and DAOTA probes remain long-lived in lipid environment and are sensitive to the “fluidity” of the microenvironment. Lastly, we were able to time gate the intrinsic nanosecond cellular fluorescence to obtain significantly improved background free FLIM images. The combination of 20 ns lifetime and ability to time gate common cellular auto fluorescence suggests these probes should be also very useful in anisotropy-based assays in that environment. It should be noted that anisotropy being a ratio-metric measurement is free from many experimental artifacts. Similarly, time gating should significantly enhance FRET measurements both in isolated systems as well in cellular environment. The 20 ns lifetime will make it possible to follow much higher energy transfer efficiencies and still satisfactorily measure the resulting shortened donor lifetime.

## Supporting Information

Text S1 **Synthesis of DAOTA butyric acid.**
(DOCX)
