# Supplementary material for: Long-Lived Bright Red Emitting Azaoxa-Triangulenium Fluorophores
Source: PLoS One. 2013 May 7;8(5):e63043. doi: 10.1371/journal.pone.0063043 (PMC3646960; doi:10.1371/journal.pone.0063043)
Supplement: Text S1 — Synthesis of DAOTA butyric acid. (DOCX) [file pone.0063043.s001.docx]

**SUPPLEMENTARY MATERIAL**

**Text S1. Synthesis of DAOTA butyric acid:**

**Preparation of DAOTA Butyric Acid Derivatives:** The synthesis of DAOTA butyric acids was an adaptation of method described earlier (1). DAOTA butyric acid tetrafluoroborate was prepared from N-(ω-(methyl butanoate)-1,8,2’,6’-tetramethoxy-9-phenylacridinium tetrafluoroborate (see reference 2 for preparation) as follows.

**N-(ω-butanoic acid)-N’-methyl-1,13-dimethoxy quinacridinium tetrafluoroborate :** 2 g (3.6 mmol) TJ354, 50 ml 33 % methylamine in ethanol and 50 ml acetonitrile is mixed and heated on a 55 °C oil bath for 5 days. The solvent was removed and 100 ml 1 M KOH added, this solution was heated on a 120 °C oil bath for 3 hours. After the reaction mixture had cooled, it was neutralized with 50% HBF_4_ (aq) and the crude product was collected by filtration. The crude was taken up in a minimum of acetonitrile, filtered and precipitated with ether (800 ml). The product (1.71 g, 3.3 mmol, 92 %) was collected by filtration as dark blue needle crystals. ^1^H NMR (DMSO) δ 12.42 (bs, 1H), 8.26 (t, *J* = 8.6, 1H), 7.96 (m, 2H), 7.84 (d, *J* = 8.8, 1H), 7.75 (d, *J* = 8.6, 1H), 7.67 (d, *J* = 8.6, 1H), 7.60 (d, *J* = 8.8, 1H), 7.03 (m, 2H), 4.66 (m, 2H), 4.16 (s, 3H), 3.73 (s, 3H), 3.72 (s, 3H), 2.68 (m, 2H), 2.17 (m, 2H). ^13^C NMR (DMSO) δ 174.2, 159.1, 158.7, 142.4, 141.6, 139.1, 138.0, 137.2, 137.0, 136.5, 118.5, 112.5, 112.3, 108.0, 107.5, 105.4, 104.8, 103.2, 103.0, 55.68, 37.42, 30.16, 20.85. MALDI-TOF MS m/z = 429. ESI-TOF HRMS-MS Calcd. for C_26_H_25_N_2_O_4_: m/z = 429.1815. Found: m/z = 429.1813

**N-(ω-butanoic acid)-N’-methyl-1,13-dimethoxy quinacridinium diazaoxatriangulenium tetrafluoroborate**: 15 g of pyridinium hydrochloride was melted at 200 °C. 300 mg TJ318 (0.58 mmol) was added and the reaction was heated to the point where the pyridinium hydrochloride starts subliming. At this point the reaction is stirred for 30 minutes. The product was precipitated with 0.2 M NaBF_4_ (aq) and collected by filtration. The dark red product (170 mg, 0.36 mmol, 62 %) was washed repeatedly with water. ^1^H NMR (CDCl_3_) δ 8.33 (t, *J* = 8.6, 1H), 8.16 (t, *J* = 8.6, 1H), 8.15 (t, *J* = 8.8, 1H), 7.79 (d, *J* = 8.8, 1H), 7.72 (d, *J* = 8.6, 1H), 7.60 (d, *J* = 8.8, 1H), 7.51 (d, *J* = 8.6, 1H), 7.40 (dd, *J* = 8.2, 2H), 4.66 (m, 2H), 4.11 (s, 3H), 2.72 (m, 2H), 2.25 (m, 2H). ^13^C NMR (CDCl_3_) δ 174.8, 152.7, 147.3, 141.7, 140.3, 140.16, 139.0, 138.8, 134.3, 133.1, 115.2, 109.1, 109.0, 108.6, 107.7, 106.0, 106.0, 101.1, 95.60, 47.4, 35.32, 29.91, 20.30. MALDI-TOF MS m/z = 383. ESI-TOF HRMS-MS m/z = 383. ESI-TOF HRMS-MS Calcd. for C24H19N2O3: m/z = 383.1396. Found: m/z = 383.1392

1. Laursen BW, Krebs FC (2001) Synthesis, structure, and properties of azatriangulenium salts. Chem Euro J 7: 1773-1783.
2. Rich RM, Stankowska DL, Maliwal BP, Sorensen TJ, Laursen BW, et.al. (2013) Elimination of autofluorescence background from fluorescence tissue images using time-gated detection and the AzaDiOxaTriAngulenium (ADOTA) fluorophore. Analyt Bioanalyt Chem 405: 2056-2075.
